# p73 is required for appropriate BMP-induced mesenchymal-to-epithelial transition during somatic cell reprogramming

**DOI:** 10.1038/cddis.2017.432

**Published:** 2017-09-07

**Authors:** Marta Martin-Lopez, Laura Maeso-Alonso, Sandra Fuertes-Alvarez, Diego Balboa, Virginia Rodríguez-Cortez, Jere Weltner, Inmaculada Diez-Prieto, Andrew Davis, Yaning Wu, Timo Otonkoski, Elsa R Flores, Pablo Menéndez, Margarita M Marques, Maria C Marin

**Affiliations:** 1 Instituto de Biomedicina (IBIOMED) and Departamento de Biología Molecular, University of León, University of Leon, Campus de Vegazana, Leon, Spain; 2 Research Programs Unit, Molecular Neurology, Biomedicum Stem Cell Center, University of Helsinki, Haartmaninkatu 8, Helsinki, Finland; 3 Josep Carreras Leukemia Research Institute, Department of Biomedicine. School of Medicine, University of Barcelona, Casanova 143, Barcelona, Spain; 4 Departamento de Medicina, Cirugía y Anatomía Veterinaria, University of León, Campus de Vegazana, León, Spain; 5 Department of Molecular Oncology, Moffitt Cancer Center, 12902 Magnolia Drive, Tampa, FL, USA; 6 Institució Catalana de Recerca i Estudis Avançats (ICREA), Barcelona, Spain; 7 Centro de Investigación Biomédica en Red de Cáncer (CIBERONC), ISCIII, Madrid, Spain; 8 Instituto de Desarrollo Ganadero and Departamento de Producción Animal, University of León, Campus de Vegazana, León, Spain

## Abstract

The generation of induced pluripotent stem cells (iPSCs) by somatic cell reprogramming holds great potential for modeling human diseases. However, the reprogramming process remains very inefficient and a better understanding of its basic biology is required. The mesenchymal-to-epithelial transition (MET) has been recognized as a crucial step for the successful reprogramming of fibroblasts into iPSCs. It has been reported that the p53 tumor suppressor gene acts as a barrier of this process, while its homolog p63 acts as an enabling factor. In this regard, the information concerning the role of the third homolog, p73, during cell reprogramming is limited. Here, we derive total *Trp73* knockout mouse embryonic fibroblasts, with or without *Trp53*, and examine their reprogramming capacity. We show that p73 is required for effective reprogramming by the Yamanaka factors, even in the absence of p53. Lack of p73 affects the early stages of reprogramming, impairing the MET and resulting in altered maturation and stabilization phases. Accordingly, the obtained p73-deficient iPSCs have a defective epithelial phenotype and alterations in the expression of pluripotency markers. We demonstrate that p73 deficiency impairs the MET, at least in part, by hindering BMP pathway activation. We report that p73 is a positive modulator of the BMP circuit, enhancing its activation by DNp73 repression of the *Smad6* promoter. Collectively, these findings provide mechanistic insight into the MET process, proposing p73 as an enhancer of MET during cellular reprogramming.

Embryonic stem cells (ESCs) are defined by their ability to proliferate by symmetrical cell divisions and to give raise to all specialized cell types (pluripotency).^1^ The possibility of generating induced pluripotent stem cells (iPSCs), with similar ESC-*stemness,* by the overexpression of the transcription factors *Oct4, Sox2*, *Klf4* and *c-Myc* (OSKM),^2^ has created new opportunities for developmental biology, disease modeling and regenerative medicine.^3, 4, 5^

iPSCs generation from mouse embryonic fibroblasts (MEFs) is a slow and inefficient process in which fibroblasts gradually lose their mesenchymal identity and assume an embryonic gene expression pattern. Functional genomics studies have defined three phases during fibroblast OSKM-induced reprogramming (termed initiation, maturation and stabilization), and uncovered an early mesenchymal-to-epithelial transition (MET) that marks the initiation phase,^6, 7^ which is dependent upon intrinsic BMP signaling. Indeed, BMP-SMAD signaling activation promotes iPSCs generation in the early reprogramming phase, confirming its role in the induction and maintenance of pluripotency.^8^

The MET process, a rate-limiting step during reprogramming, is tightly linked with the epithelial phenotype and the pluripotent state of iPSCs.^6, 9^ MET, as well as its reversal epithelial-to-mesenchymal transition (EMT), has roles in developmental biology and metastasis, highlighting the fact that reprogramming and tumor progression share some similarities.^10^ Consistently, reprogramming requires, like tumor progression, that successive barriers must be overcome to reach *stemness*.^11^ The nature of these barriers are not completely understood, but the involvement of tumor suppressors like p53^12, 13, 14, 15^ or Rb1^16^ as reprogramming hurdles has been reported, launching the hypothesis that tumor suppression mechanisms must be acting as blockades of cellular reprogramming, maintaining the differentiated state and genomic stability.

Members of the p53 family, comprised by the transcription factors p53, p63 and p73, share many functional properties, but also perform unique, and some time antagonist, biological functions.^17^ Inhibition of the p53 pathway increases iPSCs generation efficiency,^12, 13, 14, 15, 18^ whereas p63 has been reported to act as an enabling factor.^19^ A variety of p53-induced protective pathways impair reprogramming including, but not limited to, those involved in the regulation of cell growth, mainly by p53-downstream targets p21^Cip1^ or miR-34.^12, 20^ In addition, the p53–p21 axis can also restrain reprogramming by the inhibition of the MET.^21^ However, reports regarding p73 role in cellular reprogramming are inconclusive.^19, 22^ DNp73 overexpression was reported to increase human iPSCs generation efficiency,^22^ while other report concluded that p73 had no role in reprogramming, proposing that the DNp73 effect was due to its p53-dominant-negative effect.^19^ Here, we demonstrate that p73 deficiency impairs MEFs reprogramming efficiency by abating BMP-induced MET, even in the absence of p53. In agreement, p73-deficient iPSCs have an attenuated epithelial phenotype and alterations in pluripotency markers expression. We report, for the first time, that p73 is part of the BMP circuit, enhancing BMP signaling, at least in part, by DNp73 transcriptional repression of the *Smad*6 promoter.

## Results

### p73 is required for effective reprogramming by OSKM and OSK, independent of p53

**Figure 1:**
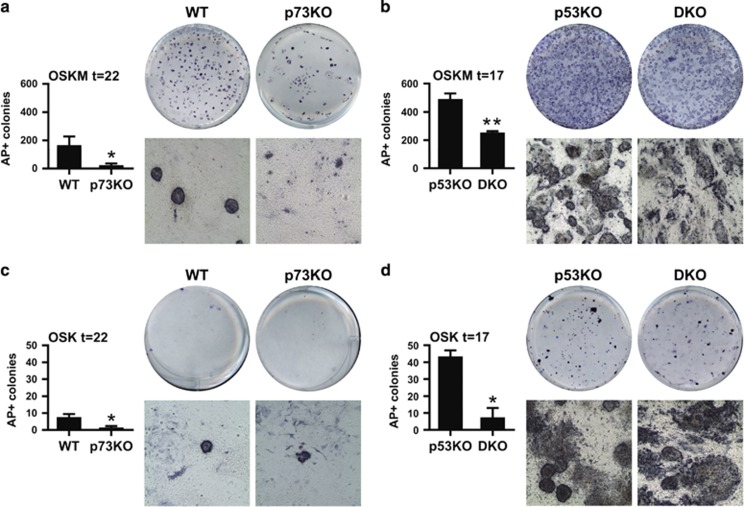
*Trp73* deficiency impairs reprogramming efficiency, even in the absence of p53. MEFs of the indicated genotypes, cultured and treated identically, were transfected with OSKM (**a** and **b**) or OSK factors (**c** and **d**) and the reprogramming efficiency was monitored by quantification of alkaline phosphatase positive colonies (AP^+^) after either 22 days for WT and p73KO (**a** and **b**) or 17 days for p53KO and DKO (**c** and **d**) of doxycycline treatment. Representative scanned plates and photomicrographs (10 ×) of the colonies are shown for each condition. Two independent reprogramming experiments were performed, including at least three biological replicates from the indicated genotypes (with the exception of p53KO-MEFs, *n*=2), with two independent transfections per replicate. Mean±S.E.M. are represented, equal variance. Student's *t*-test was performed to evaluate statistical differences; *P<0.05, **P<0.01, ***P<0.001

To analyze the effect of total p73 deficiency on reprogramming, particularly in the context of p53-loss, we sought to compare the reprogramming efficiencies of WT, p73KO, p53KO and double mutants DKO-MEFs. We transfected early passage MEFs (P4) with *piggyBac* transposon vectors encoding OSKM regulated by a doxycycline (Dox)-inducible system.^23^ Reprogramming was monitored according to previously defined morphological criteria (emergence of small cells forming compact round colonies with well-defined borders), as well as alkaline phosphatase (AP) activity.^24, 25^ After two weeks, small colonies began to appear in WT and p73KO cultures, and colonies with ESC-like morphology were collected at day 22. While WT cultures displayed typical ESC-colonies at this point, p73KO cultures exhibited a significantly lower number of irregular AP^+^ colonies (Figure 1a), indicating that lack of p73 blunted the reprogramming efficiency. Next, we addressed whether p53-induced reprogramming barriers could be accountable for the observed effect. Thus, we analyzed the expression level of *p21*^*CIP1*^ and *miR-34a.*^12, 20^ Lack of p73 neither augmented the basal expression levels of these two p53-targets in MEFs, nor its induction after 7 days of Dox-treatment (Supplementary Figure 1a).

*Trp53−/−* significantly accelerated MEFs reprogramming kinetics; however, *Trp73−/−* attenuated this p53KO-enhancing effect (Figure 1b). Lack of c-MYC delayed and attenuated WT-MEFs reprogramming^26^ and in this setting, p73KO cultures were severely affected (Figure 1c). p53 deficiency boosted OSK-reprogramming efficiency (Figure 1d), but lack of p73 also decreased p53KO-enhancing effect in these conditions (Figure 1d).

To rule out the possibility that the observed effect was due to different MEFs proliferative indexes,^27^ we analyzed growth curves from early passage MEFs littermates and found, at this early passages, no significant differences between either WT and p73KO, nor p53KO and DKO growth kinetics (Supplementary Figure 1b).

### p73 deficiency impairs MET resulting in an altered maturation and stabilization phases

**Figure 2:**
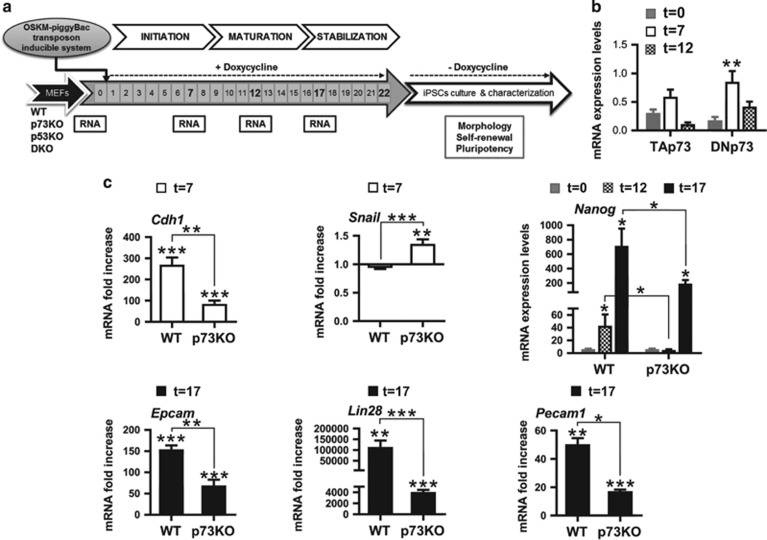
Lack of p73 impairs MET resulting in altered maturation and stabilization phases. (**a**) Overview of the experimental design of MEFs reprogramming by doxycycline inducible OSKM transfection and sample collection during the initiation, maturation and stabilization phases of the process. (**b** and **c**) Analysis of the expression kinetics profile of markers during the reprogramming process of WT (**b** and **c**) and p73KO (**c**) MEFs. RNA samples were collected at the indicated days and expression analysis was performed by qRT-PCR: (**b**) TA and DNp73; (**c**) *Cdh1*, *Snail, Nanog, Epcam*, *Lin28* and *Pecam1.* Expression of the indicated genes was normalized to 18S and set to 1 for non-transfected MEFs in each graph (*t*=0). Analysis was performed with data from two independent experiments, with at least three biological replicates from the indicated genotypes, with two replicates per sample. Mean±S.E.M. are represented, equal variance. Student's *t*-test was performed to evaluate statistical differences. *P<0.05, **P<0.01, ***P<0.001

Both *Trp73* isoforms, TA- and DNp73, were upregulated during reprogramming, being DNp73 significantly induced during the early stages of the process (Figure 2b). We used an alternative model to confirm p73 isoforms upregulation: reprogrammable-MEFs (Rep-MEFs)^28^ displayed primary mouse-ES colony-like structures 5 days after Dox-treatment and, by day 9, colonies were AP^+^ (Supplementary Figures 2a,b). qRT-PCR analysis confirmed DNp73 as the predominant isoform induced during Rep-MEFs reprogramming (Supplementary Figure 2c).

To substantiate specific p73-isoform requirement for reprogramming, we attempt to reprogram TAp73 and DNp73-specific knockout MEFs.^29^ We carry out the reprogramming experiments (Supplementary Figure 3a) and 5 days upon Sendai Virus transfection, the first mouse-ES colony-like structures began to be detected in WT and, to a lesser extent, in the TAp73-deficient MEFs (Supplementary Figures 3b,c). After 21 days, WT colonies progressed and were efficiently reprogrammed, becoming AP^+^ and expressing the pluripotency marker SSEA-1 (Supplementary Figures 3d,e). However, neither TAp73*−/−*, nor DNp73*−/−*, colonies progressed to a reprogrammed state by the end of the experiment (Supplementary Figure 3d), indicating that while DNp73 appears to be the predominantly expressed isoform, both isoforms appear to be required for the complete reprogramming.

To identify the phase of reprogramming affected by p73 deficiency, we characterized the expression kinetics of initiation, maturation and stabilization phase markers,^6^ collecting RNA samples at 7, 12 and 17 days after Dox-treatment (Figure 2a). The initiation phase (Figure 2b) is defined by the induction of epithelial-associated genes with a concomitant decrease of mesenchymal genes.^9^ Therefore, we examined the expression of epithelial markers like E-cadherin (which is upregulated during MET and crucial for reprogramming^21^) and *Epcam.* We found that in p73KO cultures upregulation of *Cdh1* or *Epcam* was significantly reduced, while the mesenchymal associated gene *Snail* showed a significant increase in the absence of p73 (Figure 2c), suggesting p73 requirement for appropriate MET establishment.

Dysregulation of MET genes impairs MEF reprogramming,^7^ thus, we speculated that in p73-deficient cells the maturation and stabilization stages would be altered. *Nanog* expression begins to increase during maturation phase^30, 31^ (Figure 2c). In agreement with an altered maturation, p73KO-iPSCs displayed attenuated *Nanog* expression and delayed kinetics profile (Figure 2d), as well as highly reduced levels of *Lin28*, a gene required for iPSCs maturation^32^ (Figure 2c). Moreover, expression of *Pecam1* was significantly decreased at the end of the stabilization phase in p73KO cells (Figure 2c), altogether demonstrating that lack of p73 impairs MET and alters the subsequent phases of the process resulting in a defective and inefficient reprogramming process.

**Figure 3:**
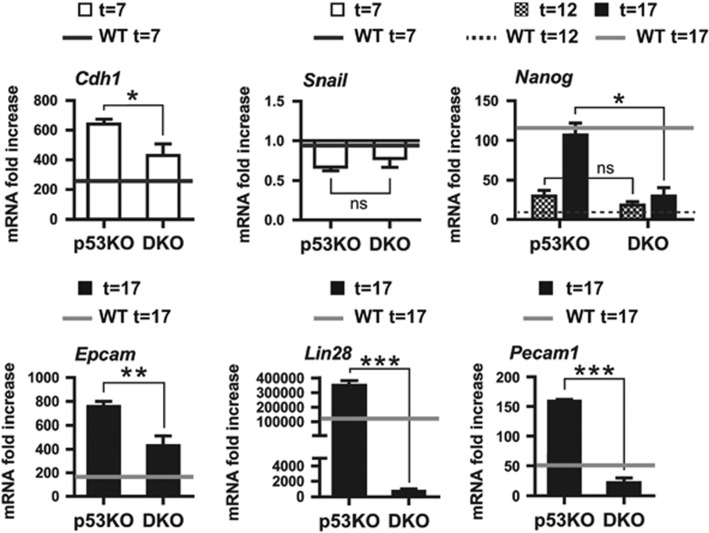
p53 deficiency could not fully counteract p73 requirement during reprogramming. Expression kinetics analysis of MET and pluripotency markers during the p53KO and DKO-MEFs reprogramming process. RNA samples were collected at the indicated days and expression analysis was performed by qRT-PCR: *Cdh1*, *Snail, Nanog, Epcam*, *Lin28* and *Pecam1.* Expression of the indicated genes was normalized to 18S and set to 1 for non-transfected MEFs in each graph (*t*=0). Analysis was performed with data from two independent experiments, with at least three biological replicates from the indicated genotypes (two biological replicates in the case of p53KO cells), with two replicates per sample. Mean±S.E.M. are represented, equal variance. Student's *t*-test was performed to evaluate statistical differences; *P<0.05, **P<0.01, ***P<0.001

*Trp53* also blocks somatic cell reprogramming by inhibiting MET through mechanisms independent of the proliferation or apoptosis pathways;^21^ thus, we sought to investigate whether p53 deficiency could counteract p73 requirement for MET induction. p53 deficiency resulted in a strong *Cdh1* and *Epcam* induction^21^ (Figure 3). Lack of p73 significantly reduced this induction despite p53 absence, highlighting p73 importance for the epithelial phenotype. However, *Trp73* loss did not affect the suppression of mesenchymal markers in this context (Figure 3), confirming that mesenchymal and epithelial transcriptional regulators are controlled by independent pathways,^9^ and that p73 regulates primarily the epithelial profile. It is noteworthy that lack of p73 had a stronger repressive effect in the induction of *Nanog*, *Lin28* and *Pecam1* expression, which levels are close to WT values in the DKO cells (Figure 3), altogether suggesting that these cells might not be attaining a fully reprogrammed state.

### p73 is dispensable for iPSC self-renewal but required for a complete reprogrammed state

**Figure 4:**
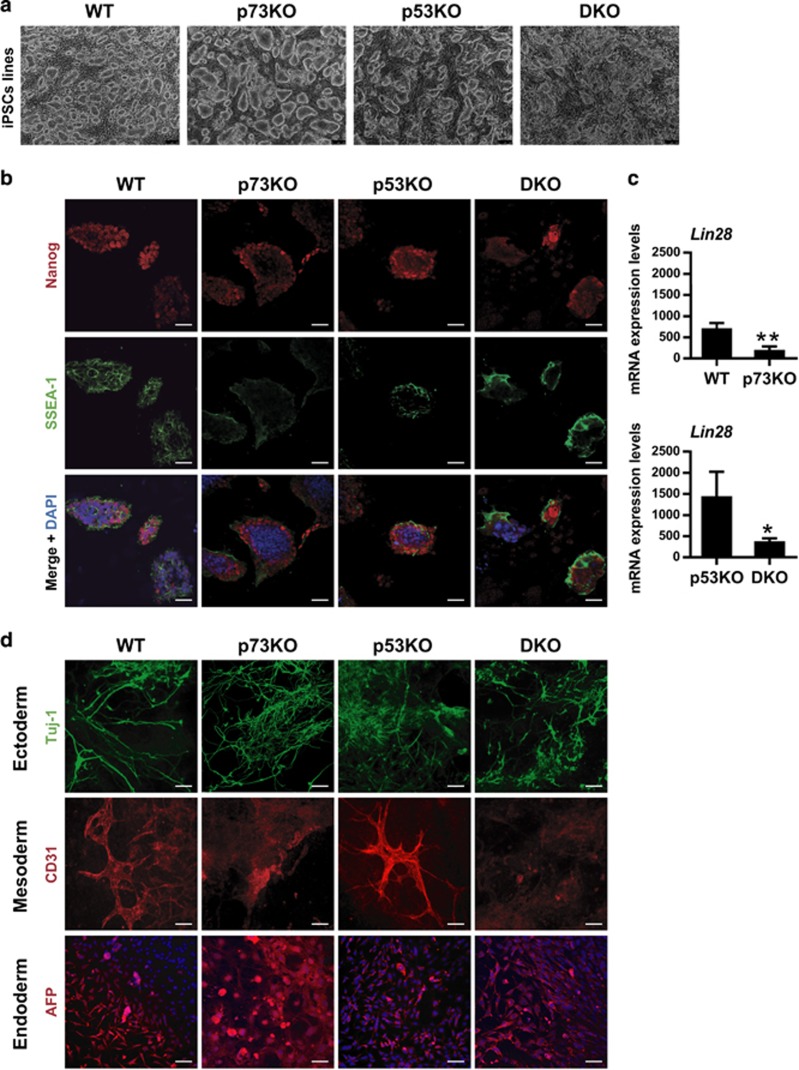
p73 is dispensable for iPSCs self-renewal and *in vitro* pluripotency, but p73-deficient cells have decreased pluripotency marker expression and might not have attained full stemness. Three clones for each genotype were analyzed. All the p73-defective clones displayed the altered phenotype (**a**–**c**) iPSCs of the indicated genotype were cultured under proliferating and non-differentiating conditions and analyzed. (**a**) Representative phase contrast images (Objective 5 × ; Scale: 250 *μ*m) of iPSCs cultures corresponding to the 20th passage. (**b**) Confocal microscopy analysis of pluripotency markers Nanog (red) and SSEA-1 (green). DAPI was used to visualize nuclei. Objective 20 ×. Scale: 80 *μ*m. (**c**) Quantification of *Lin28* expression by qRT-PCR. Mean values±S.E.M. from duplicates of at least three clones per genotype from two independent experiments are shown, equal variance. Expression was analyzed by qRT-PCR, normalized to 18S. Student's *t*-test was performed to evaluate statistical differences *P<0.05, **P<0.01, ***P<0.001. (**d**) iPSCs lines of the indicated genotype were differentiated by embryoid body (EB) formation under differentiating conditions and lineage markers of three germ layers were analyzed: Tuj-1 (ectoderm, green), CD31 (mesoderm, red) and AFP (endoderm, red). At least three clones per genotype were analyzed. DAPI was used to visualize nuclei. Objective 20 ×. Scale: 80 *μ*m

We obtained iPSCs lines by selecting primary-colonies with ESC-like morphology and expanding them by successive passages in the absence of Dox. We had severe difficulties establishing p73-deficient colonies, since many of them detached from the culture surface and did not survive after the second passage. We had only a 60%±7.14 (p73KO) and 50%±8.47 (DKO) success rate of colony establishment, compared with 90%±3.47 and 80%±3.41 for the WT- and p53KO-colonies, respectively. Nevertheless, the established colonies were grown and maintained in culture for more than 20 passages, indicating that p73 was not necessary to maintain self-renewal once the iPSC line was established (Figure 4a).

WT- and p53KO-iPSCs lines expressed key molecular markers for pluripotency, like Nanog, SSEA-1 (Figure 4b) or *Lin28* (Figure 4c). However, p73KO-iPSCs showed slightly lower levels of Nanog and SSEA-1 (Figure 4b). *Lin28* analysis revealed that p73-deficient cells did not achieve appropriate expression levels (Figure 4c), supporting the idea that these cells might not have attained full pluripotency. Nevertheless, when cultured under differentiating conditions,^33^ all derived lines showed efficient embryoid body formation and positive staining for lineage markers of the three germ layers (Figure 4c).

**Figure 5:**
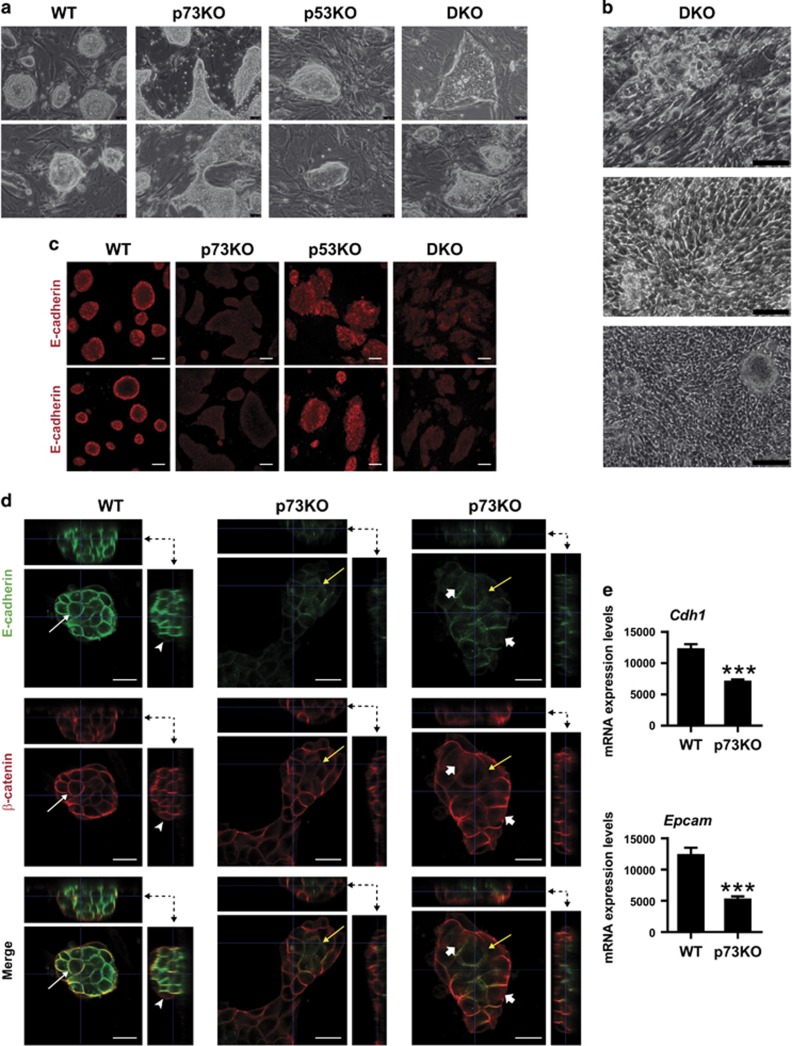
Lack of p73 results in altered iPSC colony morphology and attenuated epithelial phenotype. (**a**–**e**) iPSCs of the indicated genotype were cultured under proliferating and non-differentiating conditions and analyzed. (**a** and **b**) Representative phase contrast images of iPSCs cultures corresponding to late passages (Objective 20 × in **a** and 10 × in **b**; Scale: 250 *μ*m). Three clones for each genotype were analyzed. All the p73-defective clones displayed the altered phenotype. (**c**) Confocal microscopy analysis of E-cadherin immunostaining (red) (Objective 20 × ; Scale: 80 *μ*m). (**d**) Orthogonal projections of three-dimensional reconstruction images of iPSCs immunostained with *β*-catenin (red) and E-cadherin (green) antibodies. The lateral views are indicated by the dotted arrows and indicate the layers of cells that compose the colony. Co-localization of *β*-catenin and E-Cadherin at the plasma membrane in tight cell–cell interactions is indicated by white arrows, and cell with no co-localization at the plasma membrane are marked with arrowheads. In p73KO cells with diffuse distribution of E-Cadherin and cytoplasmic *β*-catenin are marked by arrowheads and cell without E-Cadherin/*β*-catenin interactions by yellow arrows. Objective 63 ×. Scale: 20 *μ*m. (**e**) Quantification of *Cdh1* and *Epcam* expression by qRT-PCR. Mean values±S.E.M. from duplicates of at least three clones per genotype (two biological replicates in the case of p53KO) from two independent experiments are shown, equal variance. Student's *t*-test was performed to evaluate statistical differences *P<0.05, **P<0.01, ***P<0.001

Colony morphology is one of the criteria to identify bona-fide ESC. In this regard, WT- and p53-iPSC colonies had the expected refractive appearance with tight and well-defined borders. However, p73KO-iPSC colonies, and DKO, were less compact, and had polygonal morphology and diffuse borders (Figure 5a). Moreover, at late passage, some of the DKO-iPSC lines lost mESC-colony morphology (Figure 5b), suggesting that they were not stable. In agreement, p73-deficient iPSCs had reduced levels of E-Cadherin (Figure 5c), crucial for colony compaction and full pluripotency maintenance.^34^ Orthogonal projections of confocal microscopy images confirmed that WT-iPSC colonies were dome-shaped, with several layers of cells (Figure 5d, dotted arrows) in which E-Cadherin was sharply localized at the plasma membrane (white arrows). To the contrary, p73KO-colonies were almost flat (1-2 cell layers) and had lower and diffuse expression of E-Cadherin. Reports have shown that *β*-catenin localization is a determinant factor of the pluripotent capacity.^35^ In accordance, *β*-catenin colocalized with E-Cadherin at the plasma membrane in the WT cells from the colony center that were establishing tight cell–cell interactions (Figure 5d, white arrows), but not in cells at the edge of the colony (arrowheads) which are prone to differentiate.^35^ p73KO cells had a diffuse distribution of E-Cadherin through the plasma membrane and displayed abundant cytoplasmic *β*-catenin (short arrows), indicating that E-Cadherin/*β*-catenin interactions were lost in many p73KO cells (yellow arrows). mRNA expression analysis of *Cdh1* and *Epcam* confirmed significantly reduced levels of these epithelial genes in p73-deficient cells (Figure 5e), altogether indicating that lack of p73 resulted in an impaired epithelial phenotype. Thus, our data suggest that p73 deficiency leads to defects on MET establishment during the initiation phase of OSKM-induced reprogramming, which not only result in decreased reprogramming efficiency, but also lead to alterations in the establishment of cell junctions involving E-cadherin.

### p73 is a positive modulator of the BMP circuit required for the OSKM-induced BMP signaling during the initiation phase of reprogramming

**Figure 6:**
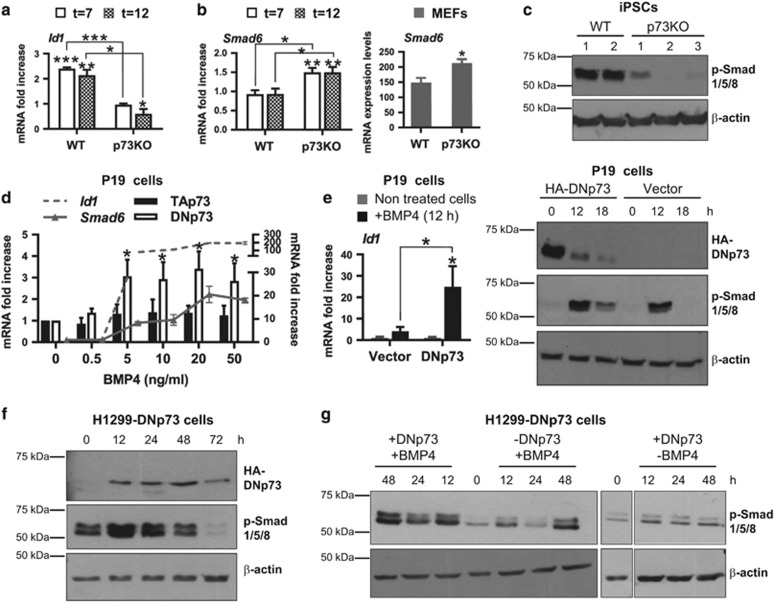
p73 is a positive modulator of the BMP circuit required for the OSKM-induced BMP signaling during the initiation phase of reprogramming. (**a** and **b**) Analysis of the expression kinetics profile of markers during reprogramming process of WT and p73KO-MEFs. RNA samples were collected at the indicated days and expression analysis was performed by qRT-PCR: (**a**) *Id1* (**b**) *Smad6* (left panel). Expression of the indicated genes was normalized to 18S and set to 1 for non-transfected MEFs in each graph (*t*=0). (**b**) Quantification of *Smad6* expression by qRT-PCR in MEFs (right panel). Expression was normalized to 18S. Analysis was performed with data from two independent experiments, with at least three biological replicates from the indicated genotypes, with two replicates per sample. Mean±S.E.M. are represented, equal variance. Student's *t*-test was performed to evaluate statistical differences. *P<0.05, **P<0.01, ***P<0.001. (**c**) Western blot analysis of BMP signaling cascade activation in WT and p73KO-iPSC clones. (**d**) Quantification of *Id1*, *Smad6*, TAp73 and DNp73 expression by qRT-PCR after BMP4 treatment (0–50 ng/ml BMP4) in serum-deprived P19 cells. (**e**–**g**) P19 cells were transfected with DNp73 expression plasmid and after 18 h, cells were serum-deprived for 24 h and then, treated with BMP4. At the indicated time points BMP cascade activation was analyzed by quantification of *Id1* by qRT-PCR (**e**, left panel) or phospho-Smad1/5/8 expression by western blot assay (**e**, right panel). (**f** and **g**) The Tet-OFF inducible cell line H1299-DNp73 was cultured on serum conditions (**e**) or serum-deprived (**g**) before inducing DNp73 expression in the presence or absence of BMP4 (**g**). BMP cascade activation was analyzed by phospho-Smad1/5/8 expression by western blot assay. Equal amounts of total protein were loaded and *β*-actin serves as loading control. Note that HA (Y11) antibody detects the exogenous DNp73 protein expression. At least two independent experiments were performed with similar results

Next, we sought to identify the mechanism underlying p73 requirement for MET establishment. MET induction is dependent upon the intrinsic activation of BMP cascade.^6^ We investigated if p73 deficiency affected OSKM-activation of BMP signaling by measuring *Id1* expression, the immediate early BMP response gene.^36^ As expected, WT cells underwent a 2.4-fold increase in *Id1* expression after 7 days of Dox-treatment, but no *Id1* induction was detected in p73KO cells (Figure 6a), suggesting a role of p73 as a BMP signaling modulator. BMP signal is fine-tuned by feedback mechanisms orchestrated, among others, by the intracellular Smad inhibitor, Smad6.^37^ We observed a significant induction of *Smad6* expression in p73KO cells when compared with WT cells (Figure 6b, left panel). Accordingly, higher levels of *Smad6* were detected in p73KO-MEFs (Figure 6b, right panel). Moreover, BMP signaling cascade activation, which was measured by the levels of phosphorylated-Smad1/5/8, was remarkably reduced in the obtained p73KO-iPSC clones (Figure 6c).

We evaluated p73 expression after BMP4 treatment of serum-deprived embryonal carcinoma P19 cells, known to upregulate *Smad6* in response to BMPs.^37^ As depicted in Figure 6d, BMP-induced DNp73 levels, but not TAp73, correlated with increased levels of other BMP targets like *Id1* or *Smad6*. Moreover, ectopic expression of DNp73 in serum-deprived P19 cells, enhanced BMP4-induced *Id1* expression (Figure 6e, left panel) and prolonged Smad1/5/8 phosphorylation (Figure 6e, right panel). DNp73 role as an enhancer of BMP signaling was also investigated in the Tet-OFF inducible cell line H1299-DNp73.^38^ In this system, reported to secrete low levels of endogenous BMP4,^39^ cells cultured in the presence of serum, but without BMP4 treatment, displayed detectable levels of p-Smad-1/5/8 that were enhanced upon induction of DNp73 expression (Figure 6f). Moreover, in serum-deprived conditions, DNp73 expression enhanced BMP4-induced Smad1/5/8 activation (Figure 6g), demonstrating that DNp73 expression potentiated BMP signaling cascade.

### DNp73 directly binds the *SMAD*6 promoter and represses its BMP-induced activation

**Figure 7:**
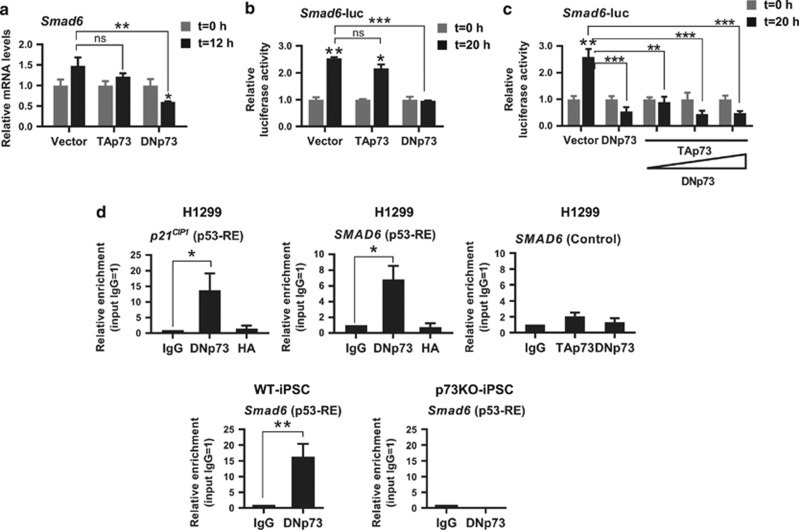
Smad6 is a direct DNp73 transcriptional target. (**a–c**) P19 cells were transfected with either (**a**) TA-, DNp73 or vector control, or (**b**,**c**) *Smad6*-promoter reporter system. After 18 h cells were serum-deprived for 24 h and then treated with BMP4. At the indicated time points samples were collected and analyzed. (**a**) Quantification of *Smad6* expression level was analyzed by qRT-PCR. (**b** and **c**) Transcriptional analysis was performed with the reporter vector pGL2-m*Smad6*-promoter (-3123)-*luc,* a BMP-responsive reporter construct that includes the p53-RE, together with the indicated expression vectors in the presence or absence of BMP4. (**c**) TAp73 (0.2 ug) was co-transfected with increasing amounts of DNp73 (0.2–0.6 *μ*g) before BMP4 treatment. Luciferase activity was normalized by the Renilla activity of the same lysate. Bars represent mean values±S.E.M of at least four experiments; ns: not significant. (**d**) ChIP analysis of H1299 cells cultured in 10% serum conditions were performed using isotypic-antibody (rabbit IgG), anti-HA or anti-DNp73 specific antibodies. Real-time PCR using specific primers to amplify p53-RE of the human or mouse *Smad6* promoter, or p53-RE in the *p21*^*Cip1*^ promoter as control, were performed and the data were normalized to input chromatin samples of each case and to IgG values=1. Additional control was performed immunoprecipitating with either rabbit IgG or anti-TAp73 and anti-DNp73 specific antibodies, and PCR amplifying the ChIP product with primers specific to a region at the vicinity of the identified p53-RE (1700 bp downstream of the p53-RE), but without homology to this site (H1299 control). Experiments were repeated four times per duplicate. Bars represent mean values±S.E.M; equal variance. Student's *t*-test was performed to evaluate statistical differences; *P<0.05, **P<0.01, ***P<0.001

DNp73 capacity to lengthen BMP cascade activation, together with the higher levels of *Smad6* detected in p73KO-MEFs, lead us to hypothesize that DNp73 might be a direct repressor of this BMP inhibitor, modulating in this way, the BMP-negative feedback loop. Supporting this idea, overexpression of DNp73, but not TAp73, significantly reduced BMP4-induced *Smad6* mRNA levels (Figure 7a), suggesting that *Smad6* could be a DNp73 transcriptional target.

Thus, to address whether *SMAD6* was transcriptionally regulated by DNp73, we performed an *in silico* prediction of p53-responsive elements within the human *SMAD6* gene using p53Family-Target-Genes data base.^40^ This analysis unveiled a p53-binding site located between nt −2769 to −2737 from the transcription start site (position +1), that was partially conserved in the murine *Smad6* promoter (Supplementary Figure 4a). We analyzed DNp73 ability to antagonize BMP4-induced activation of the *Smad6* promoter using a *Smad6*-Luc reporter^37^ in P19 cells. DNp73, but not TAp73, significantly repressed BMP4 activation of the reporter gene (Figure 7b). As TA- and DNp73 can antagonistically regulate certain genes, we investigated whether DNp73 could repress BMP4-induced *Smad6* promoter activation, in the presence of TAp73. DNp73 was capable of significantly block BMP4-induced transcriptional activation independently of TAp73 expression (Figure 7c). Thus, physiological upregulation of TAp73 during the process was not opposing DNp73 modulation of BMP cascade.

Next, we performed chromatin immunoprecipitation (ChIP) assays in H1299 cells, in 10% serum conditions to demonstrate that endogenous DNp73 could directly bind to the *SMAD6* promoter p53-RE. Cross-linked cellular extracts were immunoprecipitated and the interacting DNA was quantified by qPCR using primers specific for the p53-REs of the *SMAD6* and *p21*^*CIP1*^ promoters,^41^ and compared to IgG-pulldown as background. As expected, *p21*^*CIP1*^ showed a 13.7±5.38-fold enrichment compared to IgG (*P*=0.024; Figure 7d, left panel). More importantly, we observed a significant *in vitro* direct interaction of DNp73 with the *SMAD6* promoter p53-RE (6.80±1.73; *P*=0.004; Figure 7d, right panel), demonstrating that this gene is a direct DNp73 transcriptional target. This enrichment was not detected when ChIP was performed in a region at the vicinity of the identified *SMAD6* p53-RE but with no p53-RE homology (1700 bp downstream of the p53-RE, Supplementary Figure 4a). ChIP analysis with anti-DNp73 antibody in WT- and p73-deficient iPSCs revealed a 16.28±4.14-fold enrichment compared to IgG (*P*=0.00244; Figure 7d, lower panel) in WT-iPSCs but not in p73KO-iPSCs, further demonstrating the specificity of DNp73 direct interaction with the *Smad6* promoter.

## Discussion

Somatic cell reprogramming is an inefficient process in which successive barriers must be overcome to reach the pluripotent state.^27, 42^ It is well established that tumor suppressor genes such as *p53*, *p16*^*INK*^ or *Rb1*, that control cell proliferation, differentiation and cell death, serve as key regulators limiting cell reprogramming and maintaining cell fate and genomic stability.^43^ The members of the p53 family are transcription factors known to regulate such processes in somatic and stem cells.^17^ The emerging picture portraits an interconnected network in which p63 and p73 share many p53-functional properties, but also perform unique, and some time antagonist, biological functions.^44, 45^ Along the same line, p53 and p63 seem to play opposites roles in MEFs reprogramming. *Trp53* abatement enhances and accelerates reprogramming yielding defective iPSCs with genomic instability and *in vivo* tumorigenic potential,^21^ while *Trp63*, DNp63 in particular, has been reported to act as an enabling factor.^19^ In this regard, the information concerning p73 role in the reprogramming process is contradictory. DNp73 was reported to increase human iPSCs generation efficiency,^22^ while other study concluded that p73 has no role in MEFs reprogramming and proposed that DNp73 effect was mediated by its p53-dominant negative effect.^19^ To shed light on this issue, we reprogrammed total *Trp73* knockout MEFs, with or without *Trp53*, with the Yamanaka factors. Our data demonstrate that the reduced reprogramming efficiency of p73-deficient MEFs cannot be explained by enhanced p53 activity, as previously proposed,^19^ supporting a specific p73 function during reprogramming, independent of DNp73-dominant-negative effect over p53. Thus, while the data presented in this work is in accordance with DNp73 positive role during reprogramming, it is in contradiction with the second report.^19^ This one indicated that p73 deficiency had no effect on iPSC generation, self-maintenance or pluripotency. Nevertheless, some of our results are in partial consonance with their observations. We demonstrate that lack of p73 decreased the reprogramming efficiency; however, we obtained p73KO-iPSC clones and showed that p73 is dispensable for iPSC self-renewal. Moreover, p73KO-iPSCs could be differentiated into cells of the three germ layers, including neural progenitor cells, but a close analysis of p73KO-iPSCs revealed that p73 deficiency resulted in decreased pluripotency markers, altered morphology and attenuated epithelial phenotype.

During OSKM-mediated reprogramming, the majority of cells never complete the process, and only a small number become iPSCs.^11^ We provide evidence that the lack of p73 function reduces even further this number. In DKO-MEFs, despite the absence of p53, lack of p73 still hinders reprogramming efficiency, suggesting that p73 performs a required function that cannot be by-passed by elimination of p53 stress–response barriers. Cell growth rate is a key parameter controlling reprogramming.^27^ In this regard, reports indicated that p73-deficient MEFs had decreased S phase and their long-term cell growth rate became different from WT-cultures after 6 passages in culture,^46^ opening the possibility that the observed effect was due to differences in the MEFs proliferative index. However, in our hands, daily growth curves from early passage MEFs littermates revealed no significant differences between WT and p73KO growth kinetics. This indicates that p73 deficiency did not affect proliferating kinetics at these early passages, when the reprogramming was performed, therefore allowing the comparison of the reprogramming kinetics between WT vs. p73KO or p53KO vs. DKO. However, it is possible that, at latter passages, proliferating rates differences will begin to arise in p73-deficient MEFs.^46^ Nevertheless, in agreement with previous reports,^46^ p53 elimination accelerates proliferation dynamics (Supplementary Figure 1b, compare right column panels with left), and p53-deficient MEFs had an accelerated reprogramming kinetic respect to WT- and p73KO-MEFs.

*Trp53* role in reprogramming is not limited to the regulation of cell growth rate,^13^ but can also restrain the process by the inhibition of the MET.^21^ The BMP-induced mesenchymal-to-epithelial transition is required during the early steps of reprogramming.^6^ Here, we demonstrated that lack of p73 results in an attenuated MET transition that could account for the reduced reprogramming efficiency detected in p73-deficient cultures. We propose, for the first time, a model in which p73 is part of the BMP circuit, acting as a positive modulator of the signaling cascade, required for the OSKM-induced BMP signaling during the initiation phase of reprogramming.

DNp73 was the predominant isoform induced during the initiation stage of MEFs reprogramming. In agreement, we demonstrate that DNp73 is an enhancer of BMP cascade through direct transcriptional repression of the BMP signaling inhibitor, *Smad6*. Thus, lack of DNp73 expression during the initial steps of reprogramming would lead to increased levels of *Smad6*, which in turn, will blunt OSKM-induced BMP signaling. Consequently, MET induction would be impaired, resulting in an attenuated epithelial phenotype (with reduced *Cdh1* and *Epcam* levels) and subsequent altered maturation and stabilization phases.

BMP signaling fails to induce an epithelial phenotype in the absence of OSKM,^6^ suggesting that BMP regulation of MET is dependent on cell-intrinsic factors. Thus, it is tempting to speculate that p73, in addition to its BMP-enhancing function, could act as a pro-epithelial factor. This idea is supported by the fact that p73-deficient iPSC colonies showed altered morphology and attenuated epithelial phenotype. p73KO-iPSCs displayed defective intercellular interactions with low and diffuse expression of E-Cadherin as well as cytoplasmic localization of *β*-catenin. These intercellular interactions are crucial for colony compaction and full pluripotency maintenance.^34^ Therefore, the altered morphology of the p73-deficient colonies could reflect the link between pluripotency and the requirement for certain intercellular interactions that might be defective in the absence of p73. Supporting the idea that p73-deficient cells might not be attaining full stemness, it is noteworthy their low levels of *Lin28,* considered necessary to obtain fully reprogrammed and stable iPSCs.^32^

Taken together, our data reveal that p73 is a positive modulator of the BMP circuit, required for BMP-induced MET during somatic cell reprogramming. Our findings provide mechanistic insight into the MET regulation, supporting a specific p73 function, independent of DNp73-dominant-negative effect over p53, and highlighting the yin-yang role of the p53 family members as regulators of the reprogramming process.

## Materials and methods

### Mice husbandry and animal breeding

Animal experiments were conducted in agreement with European (Council Directive 2010/63/UE) and Spanish regulations (RD 53/2013) on the protection of experimental animals. All the protocols used within this study had the appropriate institutional committee approval. Breeding and genotyping of wild-type mice (WT), *Trp73* knockouts (p73KO), *Trp53* knockouts (p53KO) and the double mutants *Trp73−/− Trp53−/−* (DKO) were performed as described before.^47^

### Generating the TAp73^Δtd/Δtd^ mouse

The cre-loxP strategy was used to generate the TAp73 conditional knockout reporter allele (TAp73^fltd^). Genomic p73 DNA from intron 1 to intron 3 was amplified from mouse genomic DNA (C57BL/6). A neomycin resistance gene (neo) flanked by frt sites was inserted in intron 3. LoxP sites were cloned into the endogenous locus 5′ to exon 2 and 3′ of the frt-flanked neo cassette. tdTomato was cloned upstream of the 5′ loxP site and the synthetic CAG promoter was cloned downstream of the 3′ loxP site. The modified p73 locus was cloned into pL253.^48^ Mouse embryonic stem cells (G4) electroporated with the targeting vector were analyzed by Southern blot analysis for proper targeting of the TAp73 conditional knockout reporter allele. Resulting chimaeras were mated with C57BL/6 albino females and genotyped as described below. Mice with germline transmission of the targeted allele (TAp73^fltd^) were intercrossed to generate homozygous mice (TAp73^fltd/fltd^). TAp73^fltd/fltd^ mice were intercrossed with Zp3-Cre (C57BL/6) transgenic mice.^49^ TAp73^fltd/+^; Zp3-Cre mice were intercrossed to generate TAp73^fltd/Δtd^; Zp3-Cre mice, which were subsequently intercrossed to generate TAp73^Δtd /Δtd^ mice. All procedures were approved by the IACUC at University of Texas M.D. Anderson Cancer Center.

### Cell culture

Mouse Embryonic Fibroblasts (MEFs), P19 cells and H1299-DNp73*β* cells were cultured in Dulbecco's modified Eagle's medium (DMEM) supplemented with 10% fetal bovine serum (FBS) and 2 mM l-Glutamine. MEFs were maintained on 0.1% gelatin-coated plates. The Rep-MEFs, which contain a unique copy of the doxycycline inducible polycistronic cassette encoding the OSKM factors, were derived from the *in vivo* reprogrammable mice i4F^28^ and were kindly provided by Dr. Manuel Serrano (Institute for Research in Biomedicine, Barcelona, Spain). The DNp73 null MEFs^29^ and non-published TAp73Δtd/Δtd were provided by Dr. Elsa Flores (Moffitt Cancer Center, Florida, USA) and were reprogrammed using a replication-defective and persistent Sendai virus (SeV) policistronic vector encoding the OKSM genes, reported to efficiently reprogrammed MEFs.^50^

P19 mouse embryonal carcinoma cells were kindly provided by Dr. Han Li (Spanish National Cancer Research Centre, Madrid, Spain). H1299-DNp73*β* cells were a gift of by Dr Xinbin Chen (University of California, Davis, USA). These cells stably express the DNp73*β* isoform under control of a tetracycline-inducible system ‘*Tet-off”*.^38^ Cells were cultured in the presence of tetracycline (2 *μ*g/ml). When it was required, to induce DNp73 expression, cells were cultured without tetracycline. Mouse Embryonic Fibroblasts were isolated from 13.5 days *postcoitum* embryos of the above-mentioned genotypes using standard procedures. For the analysis of cell growth rates, early passage cells derived from littermates were seeded at a concentration of 10 000 cells per cm^2^ (for daily counts) or 20 000 cells per cm^2^ (for serial passages following a 3T3 protocol).

For experiments with P19 and H1299 cells involving BMP4 treatment, cells were serum-deprived (0.2% FBS) for 24 h, before the treatment with 0.5 to 50 ng/ml human BMP4 (Peprotech, Rocky Hill, NJ, USA) as indicated. Transfection of P19 cells was performed using Lipofectamine™2000 Transfection Reagent (Invitrogen, Carlsbad, CA, USA), following the manufacturer's instructions. Eighteen hours after transfection, cells were serum-deprived as described and cells were treated with 5 ng/ml BMP4.

### Reprogramming of mouse embryonic fibroblast cells and culture of iPSCs

Mouse iPSCs were generated using the *piggyBac* (PB) transposition system, as previously described.^23^ The PB transposon vectors were kindly provided by Dr Hämäläinen (Biomedicum Stem Cell Centre, Helsinki, Finland) including: (a) PB-TET-OSKM-IRES*-βgeo*, for the expression of mouse *Oct4, Sox2, Klf4* and *c-Myc* (OSKM) from a doxycycline (Dox)-inducible polycistronic construct, (b) PB-CAG-rtTA, encoding the reverse tetracycline transactivator (rtTA) and (c) pCAG-PBase, the expression vector for the transposase. Two independent reprogramming experiments were performed, each including at least three biological replicates from the indicated genotypes (with the exception of p53KO-MEFs, *n*=2), with two independent transfections per replicate.

Early passage MEFs from the indicated genotypes (WT, p73KO, p53KO and DKO) were transfected with the Neon electroporation device (ThermoFisher, MA, USA), according to the manufacturers instruction. Briefly, 200 000 cells (up to passage 4) were electroporated with 1 *μ*g PB-TET-mOSKM or PB-TET-mOSK, 0.5 *μ*g PB-rtTA and 0.5 *μ*g pCAG-PBase plasmid. Expression of the transgenes was induced the following day by treatment with 1.5 *μ*g/ml Doxycycline (Dox) in iPSC media (DMEM supplemented with 15% FBS, 2 mM l-Glutamine, 1 mM sodium pyruvate, 1 mM nonessential amino acids, 0.1 mM *β*-mercaptoethanol and 1000 U/ml leukemia inhibitory factor, LIF). Colonies were manually picked and cultured on mouse fibroblast-inactivated feeder cells in iPSC media without Dox. Alkaline phosphatase (AP) staining was performed with Alkaline Phosphatase Detection Kit (Merck, Darmstadt, Germany) according to the manufacturer’s instructions. Transgene expression was confirmed in MEFS from the four genotypes by *β*-galactosidase activity (genes encoding OSKM were linked to a *lacZ* reporter).

The Rep-MEFs can be reprogrammed *in vitro* upon addition of doxycycline.^28^ Early passage Rep-MEFs-WT were seeded at 200 000 cells/well of 6-well plate. Next day, Rep-MEFs were treated with Dox at 1*μ*g/ml (in iPSC media) to activate the cassette during the whole process. Colonies appear around day 5 after treatment and were stained for AP activity at day-16^28^

TAp73 and DNp73-specific knockout MEFs^29^ were infected with the tetracistronic SeV vector encoding OKSM factors at MOI of 3. Tetracistronic SeV was developed, generated, concentrated and tittered as previously described.^50, 51^ Briefly, two days before SeV transduction, 50 000 cells were plated in 6-well plates. Then, cells were transduced with SeV vectors during 2 h at RT. Fresh media was added and cells were incubated at 37 °C o/n. Next day, 25 000 cells were harvested and plated onto MEF irradiated feeder cells with human embryonic stem cell (ESC) medium/MEF conditioned media supplemented with 8 ng/ml basic fibroblast growth factor (Miltenyi, Bergisch Gladbach, Germany). After 3 days of transduction, the culture medium was changed every other day until the analysis of reprogramming efficiency.

### *In vitro* differentiation of iPSCs

Cells were harvested and embryoid bodies (EBs) were prepared by the hanging drop procedure as previously described.^52^ Briefly, 20 *μ*l-cell suspension drops (30 000 cell/ml in iPSC medium without LIF) were cultured for 4 days hanging from the lid of a Petri dish. EBs were then flushed down with EB medium and kept in suspension in bacteriological dishes for 3 additional days. Then, EBs were transferred to 0.1% gelatin-coated tissue culture plates and media was changed every 2 days. After 15 days, cells were fixed with 4% para-formaldehyde and used for further analysis.

### Immunostaining

Immunofluorescence was performed as described.^33^ The following primary antibodies and dilutions were used: rat anti-CD31 1 : 1000 (BD, Franklin Lakes, NJ, USA), mouse anti-Tuj-1 1 : 1000 (Covance, Princeton, NJ, USA), mouse anti-AFP 1 : 1000 (Inmunostep, Salamanca, Spain), rabbit anti-Nanog 1:1000 (Chemicon, Billerica, MA, USA), mouse anti-SSEA-1 (MC-480) 1 : 100 (Pierce, Waltham, MA, USA), rabbit anti-E-cadherin 1 : 60 (Cell Signaling, MA, USA), mouse anti-E-cadherin 1:200 (Cell Signaling, Danvers, MA, USA), rabbit anti-*β*-catenin 1 : 200 (BD). Secondary antibodies were: Alexa 647 goat anti-IgG rabbit (Molecular Probes, Eugene, OR, USA), Alexa 488 goat anti-IgG mouse (Molecular Probes), Alexa 568 donkey anti-IgG rat (Molecular Probes), Cy3 donkey anti-IgG rabbit (Jackson Immunoresearch, West Grove, PA, USA) and FITC donkey anti-IgG mouse (Jackson Immunoresearch). Images were obtained with NIKON EclipseTE2000 and ZEISS LSM 800 confocal microscope.

### RNA isolation and real-time qRT-PCR analysis

Total RNA from cultured cells was isolated using TRI reagent solution (Ambion, TX, USA) and purified with a Nucleospin RNA cleanup kit (Macherey-Nagel, Düren, Germany), according to the manufacturer’s instructions. DNase treatment was performed separately using RQ1 DNase (Promega, WI, USA) in the presence of Protector RNase inhibitor (Roche, Basel, Switzerland). First strand cDNA was synthesized using up to 2 *μ*g of total RNA and the High Capacity RNA-to-cDNA kit (Applied Biosystems, Carlsbad, CA, USA). Gene expression was analyzed by real-time qRT-PCR in a StepOnePlus Real-Time PCR System (Applied Biosystems) using FastStart Universal SYBR Green Master (ROX) (Roche). All protocols were performed according to the manufacturer's instructions. Primers sequences and conditions were described before^53^ and are indicated in the Supplementary Table 1. mRNA expression levels were calculated according to the formula: relative expression of gene= 2^(Ct internal reference-Ct gene), using 18S mRNA expression as internal reference.

### Western blot analysis

Immunoblot was performed as previously described^54^ with the following primary antibodies: rabbit anti-pSmad1/5 (Ser463/465) 1:1000 (Cell Signaling), rabbit anti-HA (Y11) 1:1000 (Santa Cruz Biotechnology, TX, USA), rabbit anti-actin (20–33) 1:10 000 (Sigma, MO, USA), followed by the appropriate HRP-conjugated secondary antibodies (Pierce). The enhanced chemiluminiscence was detected with Super Signal West-Pico Chemiluminiscent Substrate (Pierce).

### Luciferase assay

A plasmid containing a fragment of the mouse *Smad6* promoter, inserted into pGL2-Basic vector (-3231-*Luc*),^37^ was kindly provided by Dr. Kato and Dr. Miyazono (University of Tsukuba, Japan). This reporter construct contains the putative p53-RE identified in the mouse *Smad6* promoter region. P19 cells were transfected with 0.125 *μ*g of the -3123-luc-pGL2-basic, 0.0625 *μ*g of pRLNull renilla and 0.6 *μ*g of either pcDNA3-HA-TAp73α, pcDNA3-HA-ΔNp73α or pcDNA3 expression vectors, using Lipofectamine™2000 Transfection Reagent (Invitrogen) following the manufacturer's protocol. For the co-transfection experiments, P19 cells were co-transfected with a fixed amount of TAp73 (0.2 *μ*g) and different amounts of DNp73 from 0.2 to 0.6 *μ*g. Eighteen hours after transfection, cells were serum-deprived for 24 h, followed by BMP4 treatment (5 ng/ml) as described before. Luciferase activity was assayed using the Dual-Luciferase Reporter System (Promega) in a Berthold's luminometer. Firefly luciferase values were normalized to the corresponding Renilla luciferase levels.

### Chromatin immunoprecipitation

ChIP analysis was carried out as previously described.^55^ Briefly, 20x10^6^ H1299 cells, WT-iPSC or p73KO-iPSC were fixed with 1% formaldehyde for 10 min at room temperature. The reaction was stopped by addition of 0.125 mM glycine for 10 min at room temperature and cells were washed with PBS and lysed in 0.7% SDS lysis buffer. Cross-linked chromatin was fragmented by sonication to an average size of 400 bp using a Bioruptor® sonicator (Diagenode, Liege, Belgium). Chromatin was immunoprecipitated with the following antibodies: anti-p73 N terminal (Abcam, Cambridge, UK) and anti-p73 Delta N (38c674) (Abcam). Antibodies and cell lysates were incubated overnight at 4 °C before the addition of protein G-coupled magnetic beads (Dynabeads, Invitrogen) for 4 h at 4 °C. Negative controls were prepared by incubating parallel samples with non-immune rabbit IgG and anti-HA (Santa Cruz Biotechnology) antibodies.

The protein-DNA cross-links were reversed in elution buffer (1% SDS and 50 mM Tris-HCl), followed by RNase treatment overnight at 65 °C. The eluted material was incubated with proteinase K for 3 h at 45 °C, and the DNA was purified using the QIAquick PCR purification kit (Qiagen, Hilden, Germany). Real-time qPCR was performed using SYBR Green PCR kit (Bio-Rad, CA, USA) in a StepOnePlus Real-Time PCR System (Applied Biosystems). The signals were normalized to the input (non-immune rabbit IgG immunoprecipitation). Primers encompassing the p53-RE in the human SMAD6 promoter were as follows: 5′-CACTTTGGGAGGCTAGGG and 5′-CCGCCAAGTAGCTGGAAC (amplicon, 150 bp). Primers encompassing the non-specific binding site (control) in the human *SMAD6* promoter were as follows: 5′-GGACCAATCCCGACTTTACA and 5′-TAGGTGAGGGATCACGCTTT (amplicon, 218 bp). Primers encompassing the p53-RE in the mouse *SMAD6* promoter were as follows: 5′-CAGGCAGGGAACTCTTTCAG and 5′-GTAGCTGGCAACCACCATTA (amplicon, 152 bp).

### Sequence analysis and statistical analyses

*In silico* prediction of putative p53-response elements (p53-RE) within the human *SMAD6* promoter was performed with the p53FamTaG data base.^40^ Sequence alignments, either between the identified p53-RE and the human *SMAD6* gene (ENSG00000137834) or the mouse *Smad6* (ENSMUSG00000036867) gene, were carried out using the ‘Pairwise sequence alignment’ (EMBOSS Needle) software, available from the European Bioinformatics Institute.

Statistical analyses were performed using the Student’s two-tailed *t*-test. Values with *P*<0.05 were considered statistically significant (*P<0.05, **P<0.01, ***P<0.005). The mean±S.E.M. of each value is represented. Figure legends include, when necessary, specific details regarding the number of replicates and independent replicates.

## Publisher’s Note

Springer Nature remains neutral with regard to jurisdictional claims in published maps and institutional affiliations.
