# Supplementary figures and images for: p73 is required for appropriate BMP-induced mesenchymal-to-epithelial transition during somatic cell reprogramming
Source: Cell Death Dis. 2017 Sep 7;8(9):e3034–. doi: 10.1038/cddis.2017.432 (PMC5636977; doi:10.1038/cddis.2017.432)

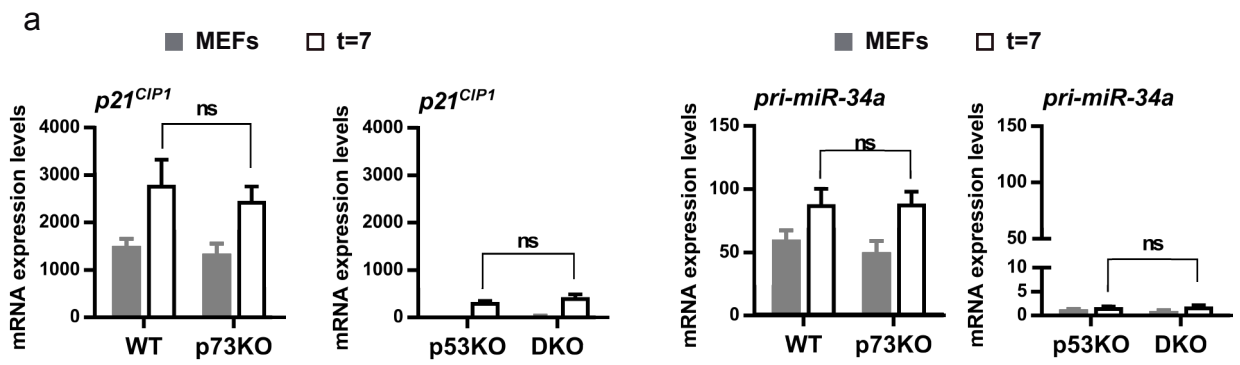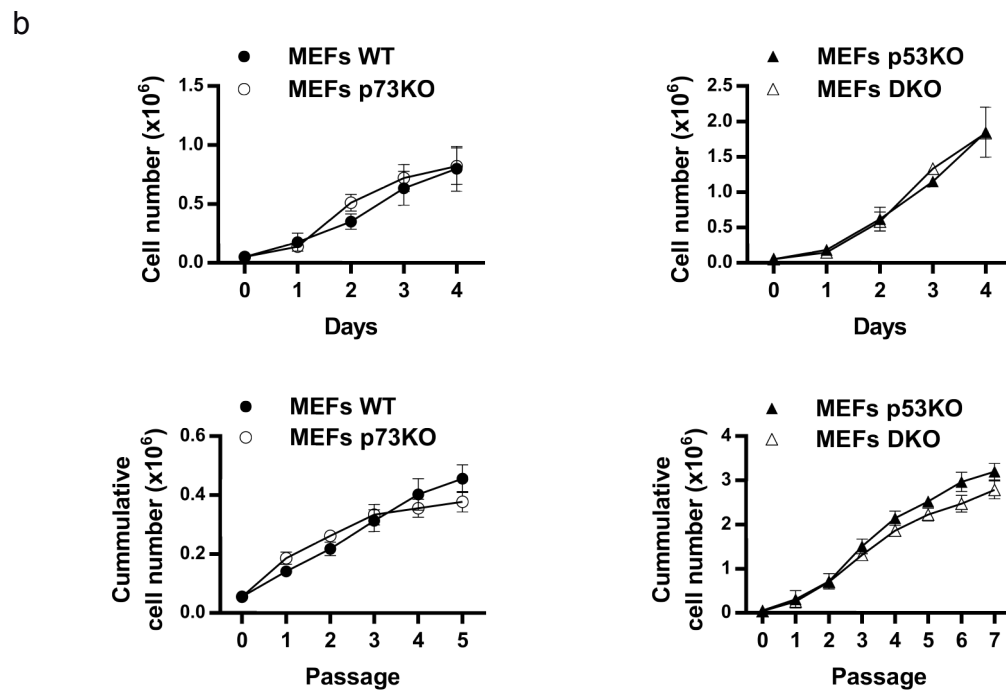

Supplementary 1. Martin-Lopez et al.

Supplement: Supplementary Figure 1 [file cddis2017432x1.pdf]

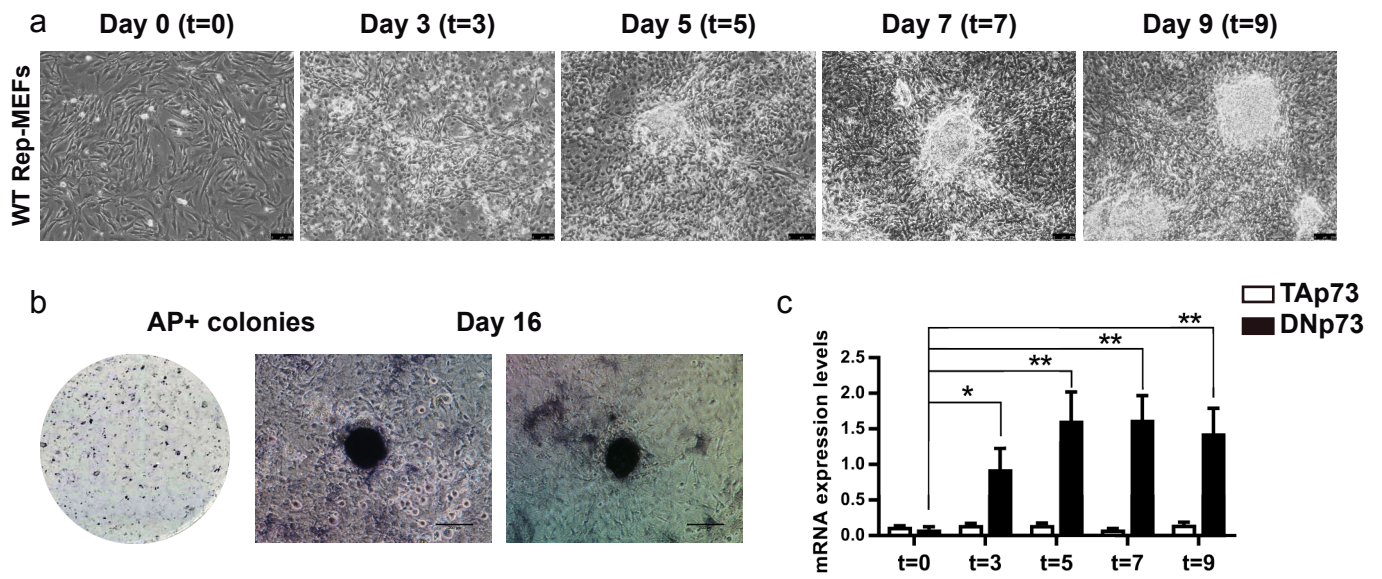

Supplement: Supplementary Figure 2 [file cddis2017432x2.pdf]

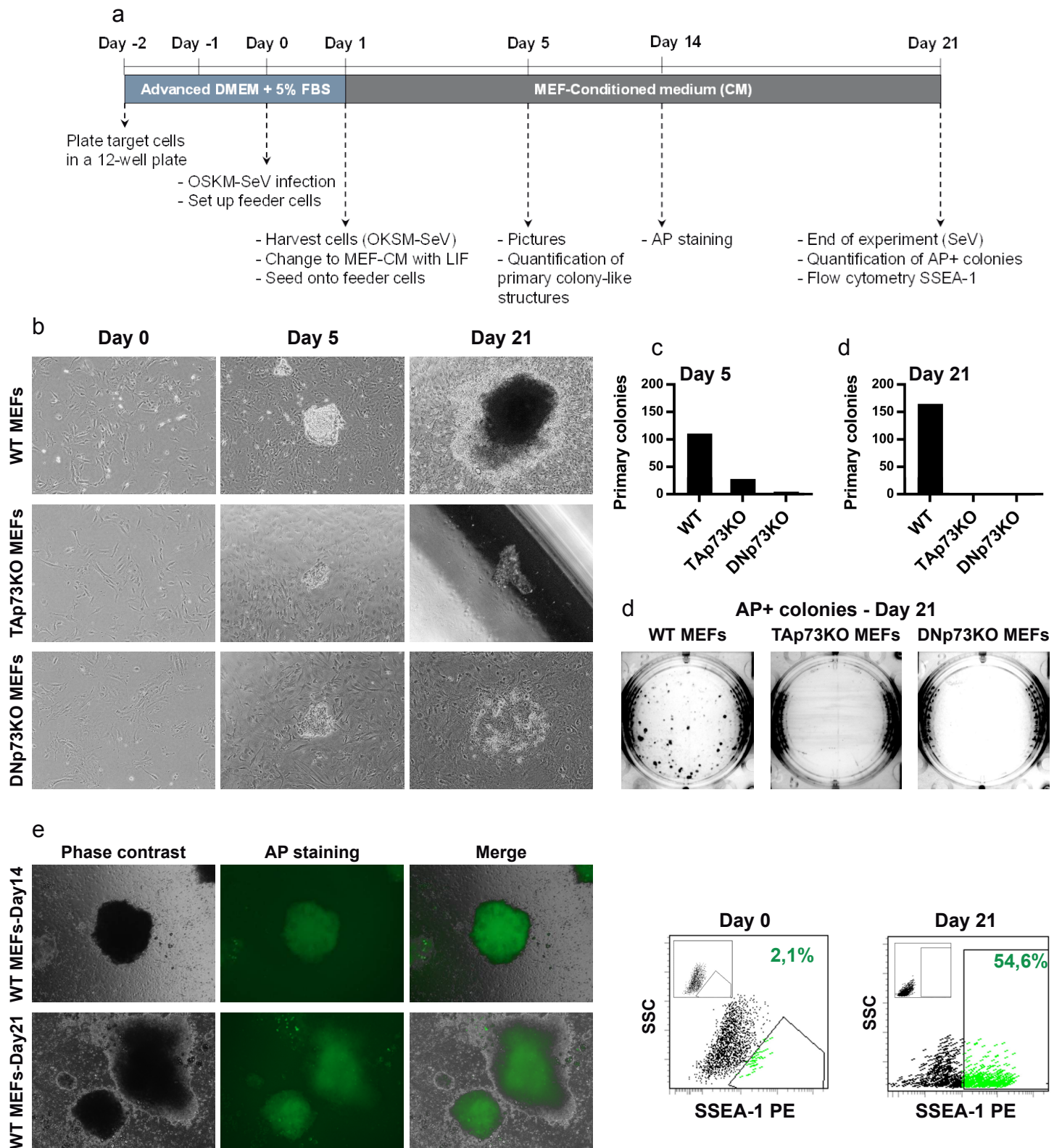

Supplement: Supplementary Figure 3 [file cddis2017432x3.pdf]
