# Supplementary material for: p73 is required for appropriate BMP-induced mesenchymal-to-epithelial transition during somatic cell reprogramming
Source: Cell Death Dis. 2017 Sep 7;8(9):e3034–. doi: 10.1038/cddis.2017.432 (PMC5636977; doi:10.1038/cddis.2017.432)
Supplement: Supplementary Figure 4 [file cddis2017432x4.pdf]

a

hSMAD6 (ENSG00000137834)

```
-3000 tgggttttaa tccggcatcg gctactaatt ataagcttgt gcaagttact tttggcaagc
      ctttgtgcct cagtgtcttt atctataaaa tggtgataat aggccggacg tggtggctca
      cacctataat cccaacactt tgggaggcta gggccggcag atctcttcag gcaggtcagg
      agttcgagac cagcctggtc aacatggtaa agccccgtct ctactaaaaa tacaaaaatt
      tgctgggcat ggtggcgcat gcctgtagtt ccagctactt ggcggggctg aggcaggaga
      atcacttgaa cccgggaggt ggaggttgca gtgagccaag atcgcaccac tgcactccag
      gctggggata gagtgagact ccgtctcaaa aaataagcaa aaaatggtgg taacaggccg
      ggcgtggtgg ctcacgctta taatcccaac actttgggag gccgaggcag gcggatcact
      tcaggcaggt caggagttcg agaccagcct ggccaacatg gtgaaaccac gtctctacta
      aaaatacaaa aattagccgg gcatagtggt gcatgcctat agttccagct atttgggagg
      ctgaggcacg agaatcactt gagtgcagag ctgagatccc accactgcac tccagtctgg
      ggacagagca aggctctgtc tcaaaaaaat aaatacatta aataaaaaat aaaaataaaa
      tggtgttaac aaccacagct atccccaaaa cgttgtattc tggggcctca acccctgaca
      ctttgattca gcaggtggac ctttgaacca cattttggaa atccctgcct aaagagtata
      gtctggcatc acagacctac acaccacttc cagccagagg atgagcaggc gtgctggttc
      atgcctctct ggttttagca tgttagcatt gtcaatgtca tcctcgtttt agtggcaagt
      tacatgagtg agctcctgtg cctcagtttt cctcatctgt aactggagat aataatagta
      actatgttaa gtccttatat attaataata atagtaccta tgttaagtgt tgtgagcgtt
      aactaagtcc tggaacattg ccttgcccat agtaacgact caatacgcac gaactattat
      tattgtaata cacagtcaaa gataatccac aaagagcata cacggggata aaagaaaata
      cactgagttt ctgtttgtat tttaagatgt attaatttcc ccatgggcga aaagtgactg
      aaaggggcca caagacaggc ttctggggtg caggtcaggt tgctttttgg gtgctggtga
      catcatgtgt gtttgttcac gtgtgaaaat tcatcctgct gttcgcttgc gatttgtgca
      tttaacgtgc actatctcca gggaccttcg tggttctgtg aagcacgaag ggcaagcatc
      ctcatccgcg gtttcaaaca agaaactgag gctaggaccg ctactttaat ttggccaaag
      tagtagggga tgggtgtgag gaagtgtctc agtccgatct ttctttcggg gagcaacggc
      cactctcaga gagcgcagcg cgaacagtac ctggttccag gtaaagctca gcaaagggtt
      ctgaatcctc cttgcaaatg ccacccttcc cccagcgcac acagactctg ggcacggccg
      ggggttcgca ccgccgcgca ctccccaggc tagctccacg cgtggtcact ctccagggag
      acccgcccgc cggcgccccg cggtcaccgc gggggaatgg cgcccagccc cgggccaatg
      agagtagcgg aatttctttc attcagcgcg gggccaggct cctgcccgga aaggggcggg
      gcgccgcgcc gggggtgggg ccgccgcgcg cggggggaga gggcggggcc ggcccctctg
      cctggccaat cggcgccgcg cccggggggg gagggggccc caggcctttt ttcccctccg
      cgcgggacca atcccgactt tacaagcttg aacttttgcc accctcggac tagcgagcgc
      gcccgaggag gtaccccgcc gcgccggctc caggggcagg agcggcctta acccttcccg
      cgccgggcgt caggctgctg ccgccgcgcc cccgatcccg cccccaaccc acccccattt
      tcgcgtggaa acctctctag ggaaagcgtg atccctcacc tacagccctc tccgaggtgc
      ggagagtaaa cttgagaagg gagaggaagc acccggcctg gagggggccc ggccggccgg
      ccccgcctct accccaccct ctccatctcc ccactccggg cgagcctgga gccaccgtac
      gcaaagcgtc ctgagctccc tgtttggccc ggcagagcga cccccgactt tgtgcaagga
      gcgtagagcc cctagtgcag tcacggaaga tccgctaccc caacatatcc gccgccccct
      gctcctcaga ggctcaaccc tggaaggcac acatgcacac ccatttttat tagcctaccc
      taggatgggg gtttggaaca actttaaagt gcaaagtgtg tttgtaagtt tgtgcacaag
      ccagggagag gcaaagcgcc ccgtttgcgt ccgagtctca gtgggtccgc cgagccttgc
      gcgcctgctc gtggctcgtg taaccctgtg tgtgtgtgtg tgtgtgtccg cgcgcgcgtg
      tgtgctcgct cgcatccgtg ctctgggcgc aacgcgaggc agaaagcggg ccgcgggcgc
      tgtcaggccc tgggcggggg tgccggggtg gtgtggggcg ggggcccgag ctcggcccgc
      cccgggagcc ggccccttgg agcctccggc cctgcaatgg gacgtgttct cctttaagag
  -60 ttaagaaact tgaaaccttg tttgcgcaac aatcagcgcc gcggagccgc caaagtgtct TSS
```

b

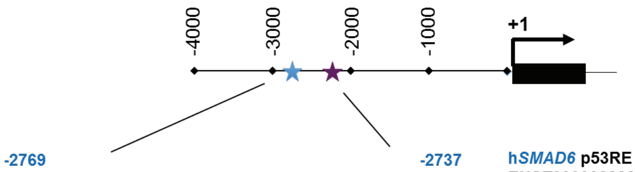


```
-2769                              -2737
 ACAAAAATTTGCTGGGCATGGTGGCGCATGCCT
 |||||..|.|||.||..||.|||.|..|.||||
 ACAAACTTGTGCAGGCTATTGTGTCCAAGGCCT
-2191                              -2159
```

h*SMAD6* p53RE (FamTaG) ★
ENST00000288840
Gene ENSG00000137834

m*Smad6* p53RE ★
ENSMUST00000041029.5
Gene ENSMUSG00000036867

Supplementary 4. Martin-Lopez et al.
