# Supplementary material for: p73 is required for appropriate BMP-induced mesenchymal-to-epithelial transition during somatic cell reprogramming
Source: Cell Death Dis. 2017 Sep 7;8(9):e3034–. doi: 10.1038/cddis.2017.432 (PMC5636977; doi:10.1038/cddis.2017.432)
Supplement: Supplementary Table 1 [file cddis2017432x5.pdf]

Supplementary Table 1. Mouse qRT-PCR primer sequences.

mRNA	Primers
**18S**	F 5´AGTTCCAGCACATTTTGCGAG 3´
	R 5″TCATCCTCCGTGAGTTCTCCA 3´
**Pecam1**	F 5″TACTGCAGGCATCGGCAAA 3´
	R 5´GCATTTCGCACACCTGGAT 3´
**Cdh1**	F 5´CCCCCTTACGACTCTCTGTTGGTGT 3´
	R 5″TCGGTTGCCCCACTCGTTCAGATA 3´
**Epcam**	F 5´GCGGCTCAGAGAGACTGTG 3´
	R 5´CCAAGCATTTAGACGCCAGTTT 3´
**Id1**	F 5´ GGCTGCTACTCACGCCTCAA 3´
	R 5´ GATCGTCGGCTGGAACAC 3´
**Lin28**	F 5´ GGCATCTGTAAGTGGTTCAACG 3´
	R 5´ CCCTCCTTGAGGCTTCGGA 3´
**Nanog**	F 5´ CTCATCAATGCCTGCAGTTTTTCA 3´
	R 5´CTCCTCAGGGCCCTTGTCAGC 3´
**p21**^{CIP1}	F 5´CCTGGTGATGTCCGACCTG 3´
	R 5´CCATGAGCGCATCGCAATC 3´
**pri-miR34a**	F 5´ CTGTGCCCTCTTGCAAAAGG 3´
	R 5´ GGACATTCAGGTGAGGGTCTTG 3´
**Smad6**	F 5´ GGCTGTCTCCTCCTGACCAGT 3´
	R 5´ CAATGTAGAATCGGACAGATC 3´
**Snail1**	F 5´ CACACGCTGCCTTGTGTCT 3´
	R 5´ GGTCAGCAAAAGCACGGTT 3´
**TAp73**	F 5´GCACCTACTTTGACCTCCCC 3´
	R 5´GCACTGCTGAGCAAATTGAAC 3´
**ΔNp73**	F 5´ATGCTTTACGTCGGTGACCC 3´
	R 5´GCACTGCTGAGCAAATTGAAC 3´
