# Supplementary material for: p73 is required for appropriate BMP-induced mesenchymal-to-epithelial transition during somatic cell reprogramming
Source: Cell Death Dis. 2017 Sep 7;8(9):e3034–. doi: 10.1038/cddis.2017.432 (PMC5636977; doi:10.1038/cddis.2017.432)
Supplement: Supplementary Figure Legends [file cddis2017432x6.docx]

**Supplementary Figure Legends**

**Supplementary Figure 1.** **The effect of p73 deficiency on reprogramming is not due to upregulation of p53-reprogramming barriers affecting cellular proliferation**. Quantification of *p21^CIP1^* and *pri-miR-34a* expression by qRT-PCR in **(a)** MEFs WT-p73KO and p53KO-DKO at passage 3, **(b)** during the reprogramming process (7 days after dox treatment) and **(c)** analysis of the cell growth and cumulative growth curve in MEFs WT-p73KO and p53KO-DKO. Analysis was performed with data from two independent experiments, with at least three biological replicates from the indicated genotypes (two biological replicates in the case of p53KO cells), with two replicates per sample. ns: not significant. Mean±S.E.M. are represented, equal-variance. Student´s-test was performed to evaluate statistical differences. *p<0.05, **p<0.01, ***p<0.001.

**Supplementary Figure 2.** Analysis of the expression kinetics profile of TA and DNp73 during the reprogramming process of Rep-MEFs WT. a) Representative phase contrast images (10X) of the reprogramming process. b) The reprogramming of Rep-MEFs WT was monitored by alkaline phosphatase activity after 16 days of doxycycline treatment. Representative scanned plate of the AP positives colonies is shown. c) Analysis of the expression kinetics profile of TA and DNp73 during the reprogramming process of Rep-MEFs WT. RNA samples were collected at the indicated times and expression analysis was performed by qRT-PCR, normalized to 18S. Each value from each genotype was compared against t=0. Mean±S.E.M. are represented, equal-variance. Student´s-test was performed to evaluate statistical differences, *p<0.05, **p<0.01, ***p<0.001.

**Supplementary Figure 3**. TAp73 and DNp73 isoforms appear to be required for the complete reprogramming process. **a)** Diagram of the experimental design of WT, TAp73 and DNp73-specific knockout MEFs reprogramming by a Sendai virus policistronic vector encoding the OKSM genes. **b)** Representative phase contrast images (4X) of the colonies are shown during the indicated times of the process. **c-d)** Quantification of primary mouse-ES colony-like structures after 5 and 21 days upon SeV transfection and **(c)** representative scanned plates of the AP positives colonies at 21 days upon SeV transfection. **d)** Representative phase contrast and AP staining image (green) (Objective 4X) after 14 and 21 days upon SeV transfection and flow cytometry analysis of the pluripotency marker SSEA-1 (green) in WT- MEFs after 21 days upon SeV transfection.

**Supplementary Figure 4**. **a)** *In silico* prediction of p53-responsive element within the human *SMAD6* gene, using p53Family-Target Genes data base, unveiled a p53-binding site located between nt -2769 to -2737 (red in bold), from the transcription start site (position +1). Primers sequences encompassing the p53-RE in the h*SMAD6* promoter are highlighted in green, while sequences encompassing a region located 1700 pb downstream from de p53-RE with no recognized p53 response element are in blue. **b)** Pairwise alignment between human and mouse *SMAD6* promoter sequences unveiled a partially conserved p53-RE in the murine *Smad6* promoter. TSS: transcription start site.
